# Sifting and Winnowing through Human Cytomegalovirus Lytic Replication and Latency

**DOI:** 10.1371/journal.ppat.1005607

**Published:** 2016-09-22

**Authors:** Robert F. Kalejta

**Affiliations:** Institute for Molecular Virology and McArdle Laboratory for Cancer Research,University of Wisconsin-Madison, Madison, Wisconsin; The Fox Chase Cancer Center, UNITED STATES

My love of nature began in the field, forest, and creek behind my house where I spent my summer days catching butterflies, salamanders, minnows, and toads. In high school, the logic and irrefutability of geometry proofs turned me into a rational thinker. College steered me towards molecular biology. Graduate school introduced me to the art of generating data. My mentor, Joyce Hamlin, and the great members of her lab taught me the scientific method, the importance of controls, and how to effectively communicate my results both in writing and in talks. I learned you can’t troubleshoot experiments unless you know how they are supposed to work (don’t just follow the protocol, but really understand it), and that scientific discovery requires hard work and long hours. My postdoctoral work made me a virologist. My mentor, Tom Shenk, taught me there are no excuses for not doing the right experiment. The diverse and eclectic lab group he assembled taught me that listening to others’ work and opinions can help move your own project forward. My failed fellowship applications, delayed job search, and advice from local virology luminaries taught me that work doesn’t count unless it’s published. Finally, landing a job at a virology institute without ever doing an experiment in an infected cell and at a cancer research lab working on a virus that (probably) does not cause cancer taught me that limits are things you put on yourself, and that it doesn’t hurt to try.

Our lab studies two major aspects of human cytomegalovirus (HCMV) biology, the first of which is the interplay between viral and cellular proteins that control cell cycle progression. Our work has shown that HCMV has multiple strategies to modify a key cellular tumor suppressor in a way always described as inactivation, yet the protein is paradoxically required for efficient viral infection. During these investigations, we identified a new class of viral proteins able to modulate cellular tumor suppressors. The work is important because it is revealing key similarities and differences between how cellular tumor suppressor and cell cycle pathways are modified in virally infected or cancerous cells. The hope is that the molecular differences can be exploited for anti-viral or anti-cancer therapeutics.

The second focus of our lab is the transcriptional control of lytic and latent infections. Our team has shown that, upon entering the nucleus, infecting viral genomes are transcriptionally silenced by cellular defense proteins. The virus rapidly neutralizes this defense in certain cell types to initiate the productive replication that allows for viral spread. In other cell types, the virus actually uses the cellular defense to hide from immune surveillance by establishing latency. During these investigations, we identified the first molecular mechanisms used by HCMV to achieve and maintain latency. The work is important because it revealed that “arms races” between cells and viruses occur not only over productive (lytic) infections but over latent colonization as well. The hope is that molecular details of HCMV latency will sprout control measures targeting latent reservoirs. Specifics about all our work can be found on our lab web site (http://kalejta.virology.wisc.edu/).

I like to think our research matters on multiple levels, and in different ways to different groups of people. Our work has scientific impact because it explores basic principles behind fundamental biological processes yet also has clinical implications. It delivers information about a pathogenic human virus as well as general cell biology, doubling the return on precious investments of effort and money. Our research matters because it promotes the very idea that accumulating knowledge and seeking the truth are worthwhile goals. I hope it serves as a beacon to the community as our state suffers under a political climate that stifles academic freedom and the search for truth through the time-honored process of “sifting and winnowing.” Our research matters because it has a strong economic impact in the local community, generating literally millions of dollars in outside funding, a large fraction of which returns to local businesses. The grant money we receive and pay out in salaries has helped my students and postdocs start careers and families, become United States citizens, and pursue their own goals and dreams.

Our research matters because it is my legacy to the future, my attempt to leave the world a little bit better than how I found it. It’s a lot of fun, and a new challenge everyday. The changing face of an academic lab introduces me to new people from all over the country and the world, and keeps me young at heart. Despite the constant struggle for funding, it’s a privilege to do something important that you love, and to get paid for doing it. In a time when students are bombarded with information about “alternative careers,” we should also be reminding them of the pleasures and rewards of academic research.

**Image 1:**
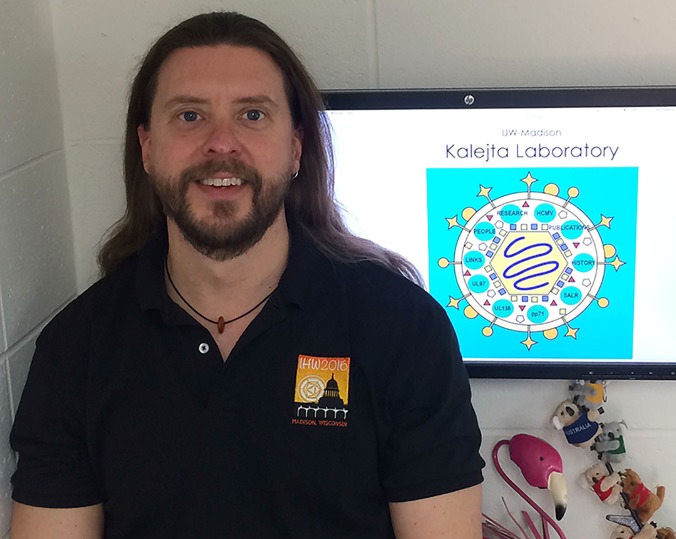
Robert F. Kalejta.

